# BCG-Induced Immune Training: Interplay between Trained Immunity and Emergency Granulopoiesis

**DOI:** 10.1016/j.jmb.2023.168169

**Published:** 2023-05-30

**Authors:** Henok Andualem, Elysia Hollams, Tobias R. Kollmann, Nelly Amenyogbe

**Affiliations:** 1 - Department of Medical Laboratory Science, College of Health Science, Debre Tabor University, Ethiopia; 2 - Telethon Kids Institute, Perth, Western Australia, Australia

**Keywords:** Emergency granulopoiesis, trained Immunity, mechanism, modulators, BCG

## Abstract

Bacille Calmette-Guérin (BCG) is the most commonly administered vaccine in human history. The medical application of BCG extends far beyond the fight against tuberculosis. Despite its stellar medical record over 100 years, insight into how BCG provides this vast range of benefits is largely limited, both for its pathogen-specific (tuberculosis) as well as pathogen-agnostic (other infections, autoimmunity, allergies, and cancer) effects. Trained immunity and emergency granulopoiesis have been identified as mediating BCG’s pathogen-agnostic effects, for which some of the molecular mechanisms have been delineated. Upon review of the existing evidence, we postulate that emergency granulopoiesis and trained immunity are a continuum of the same effect cascade. In this context, we highlight that BCG’s pathogen-agnostic benefits could be optimized by taking advantage of the age of the recipient and route of BCG administration.

## Introduction

An effective immune response to infection or vaccination results from the combined impact on innate and adaptive immunity. For many years it was assumed that immunological memory, an evolutionary trait that refines host survival upon subsequent infection, is a distinct feature of the adaptive arm of the immune system. However, among plants and invertebrates that lack adaptive immunity, primary infection induces resistance to infection from related/unrelated pathogens, highlighting the existence of immune memory other than that provided by the adaptive immune system.^1^ There is growing evidence from both animal and human studies, following initial exposure to a given pathogen that innate immune memory impacts subsequent exposure.^2–6^ Given the lack of antigen-specificity in innate immune response, such innate immune memory has been labeled “trained immunity” to differentiate it from classical adaptive immune memory. Importantly, trained immunity is pathogen-agnostic, i.e., it can potentially protect the host against a wide spectrum of pathogens; trained immunity thus improves overall host resilience to infectious threats. In humans, pathogen-agnostic protection has been well described following the administration of the live attenuated Bacille Calmette-Guérin (BCG) vaccine. Beyond its target pathogen *Mycobacterium tuberculosis* (Mtb), BCG induces host protection against diseases such as malaria (caused by *plasmodium* species), yellow fever (caused by yellow fever virus), and systemic infections by pathogenic fungi such as *C. albicans*.^7–11^ Pathogen-agnostic protective effects have also been documented following other vaccines including measles and oral polio vaccines (OPV).^5,6^

Despite the success of vaccination, not all pathogens are currently covered by existing vaccines, thus susceptibility to infection and death, especially during the neonatal period remains a significant health problem.^12^ The ability of some pathogens to cause infection before adaptive immunity has been measurably induced following vaccination (~2 weeks post-vaccination) has added another hurdle to reducing infectious mortality, again, especially among neonates.^12^ For newborns in particular, rapid and broad protection following vaccination would thus be ideal. In this regard, BCG vaccination at birth has been shown to diminish all-cause mortality in high-mortality settings during the first weeks of life via pathogen-agnostic effects (also called non-specific, off-target, heterologous or secondary effects of vaccines).^13^ Mechanistically, this effect of BCG is still under active investigation. Among the possible mechanisms, protection from neonatal polymicrobial sepsis through BCG was recently shown to involve emergency granulopoiesis (EG), i.e. rapid upregulation of neutrophil production.^11,14^

Although the range of innate immune cells that can be trained has not yet conclusively been delineated, neutrophils are among trainable innate immune cells.^15,16^ Several studies reported an increase in neutrophil numbers and an increase in neutrophilbased protection via EG as contributing to TI,^11,15,16^ but a clear dissection of the mechanistic cause-effect chain involved is still missing. Specifically, it remains unknown 1) if EG and TI are related to each other; 2) whether TI induction involves the *de novo* generation of neutrophils (via EG) before inducing longer-term functional changes of neutrophils or their precursor; 3) if not, then what immunologically distinct mechanisms regulate pathogen-agnostic protection provided by EG vs TI. Importantly, neonatal BCG-induced pathogen agnostic protection (human or animal) is variable, in that some recipients develop robust TI, while others respond weakly.^8,9,17^ Furthermore, the reason why BCG protects some but not others remains unexplained.

A further important variable to consider in the quest to identify the mechanism driving EG and TI is that for BCG-immunized adult animals, route of delivery has been associated with variation in downstream TI effects.^18–21^ The mechanisms that drive such route-dependent differences in the response have not been delineated. And lastly, how age-dependent differences affect BCG’s ability to induce EG and/or TI when given via different routes has not been examined. We here contrast mechanisms related to BCG-induced TI and EG, with differences in age and the energetic state of the recipient as well as the importance of the route of delivery not only as important determinants of the final clinical outcome but also as potential tools to dissect the responsible molecular mechanisms.

## Molecular mechanism of TI and EG

### Molecular mechanism of TI

TI Both TI and EG can be initiated by similar pathways, but are characterized by distinct phenotypic effects on the host. This section describes the host phenotype associated with TI, with a focus on metabolism via epigenetic reprogramming. It is now increasingly appreciated that the remarkable intertwining between metabolic and epigenetic programs represents the molecular mechanism behind TI in bone marrow (BM) and circulating innate immune cells. The first step of innate immune cell training begins with recognition of the triggers such as BCG or bglucan by pattern recognition receptors (PRRs), such as Node-like (NOD-2) or C-type lecithin receptors (Dectin-1). Following sensing of the training stimulus, the activation of downstream signaling cascades engenders changes in intracellular metabolism to stimulate the Akt-mTOR-HIF-1α pathway.^22^ The β-glucan-Dectin-1 signaling mediated upregulation of an immune gene–priming long noncoding RNAs (IPLs), upstream master long noncoding RNA of the inflammatory chemokines locus (UMLILO) which expands the level of WD repeat-containing protein 5 (MDR5) mixed lineage leukemia protein (MLL1) complex to immune gene promotor, this in turn promotes histone 3 lysine 4 trimethylation (H3K4me3)) enrichment.^23,24^ This may represent the molecular mechanism of H3K4me3 regulation in TI. Activation of this pathway and the resultant increase in activating histone modification (like H3K4me3) upregulates glycolysis and reflects the early connection between epigenetics and metabolic systems.^25,26^ This is followed by the conversion of pyruvate (from glucose) into lactate which effluxes from the cell or to acetyl CoA, a central point where different metabolic programs including glutamine metabolism, fatty acid (FA) synthesis, and cholesterol metabolism are regulated.^27^

Under the steady-state, the tricarboxylic acid (TCA) cycle receives energy in the form of acetyl CoA and transmits it to oxidative phosphorylation (OXPHOS) for ATP synthesis. However, when exposed to TI inducers, the TCA cycle undergoes fragmentation at specific points, allowing the buildup of metabolites, which regulate and are regulated by epigenetic enzymes.^28^ Here, beyond upregulation of the glycolysis pathway, monocytes trained with β-glucan, BCG, or OxLDL exhibited enhanced oxygen consumption, indicating upregulation of the OXPHOS pathway.^29–31^ The role of OXPHOS in TI was described in primary monocytes from healthy adult volunteers, where monocytes trained by β-glucan underwent epigenetic modification via *Set7* methyltransferase, resulting in the upregulation of genes associated with the TCA cycle.^31^ Pharmacological inhibition of epigenetic reprogramming targeting ATP synthetase abolished the induction of a trained cell phenotype, providing evidence that OXPHOS-mediated energy synthesis, and not only glycolysis, is necessary for immune cells to display the TI phenotype.^29,31^ Similar associations were documented for glycolysis, where single nucleotide polymorphisms (SNP) in genes encoding for glycolysis enzymes are liked to augmentation of cytokine production in cells trained with BCG.^29^ In contrast to training by β-glucan, BCG-induced TI was disrupted when the cellular machinery required for glycolysis was suppressed, while suppressing OXPHOS did not impact the TI phenotype.^25^ The reasons behind this difference between BCG- and β-glucan-induced TI are unknown, but may involve the dose of immune stimulant used. For instance, a high dose (10 μg/mL) of β-glucan induces the classical Warburg effect (increasing glycolysis but repressing OXPHOS), while a low dose of β-glucan (1 μg/mL), BCG, or OxLDL upregulates both energy pathways.^25,29,31,32^

Enrichment of early metabolites in the process of TI includes citrate, which leaves the TCA cycle and converts into acetyl CoA, that A) serves as a substrate for acetyltransferase and increases H3K27ac to express genes that foster glycolysis; B) fuels cholesterol synthesis pathway via mevalonate accumulation, and further replenishes glycolysis via IGF-1R-Akt-mToR pathway, which is consistently observed as an important hallmark of TI; C) supports FA synthesis, which contributes less to induction of TI, but is important in secondary effector functions.^33,34^ The role of FA was explored in another instance where BCG triggers the expression of peroxisome proliferator-activated receptor gamma (PPARγ), the activation of which upregulates FA storage in the liver; however, the in vitro exposure of immune cells with BCG diminished PPARγ expression.^35–37^ The latter was associated with inflammatory macrophage phenotypes, and whether this takes place in TI or how FA metabolism impacts restimulation of trained cells needs further investigation.

Other TCA metabolites such as succinate, fumarate, and malate which are supplied from glutamate metabolism have also been shown to be critical for TI. Particularly, fumarate has been shown to bridge metabolism with epigenetic reprogramming via direct inhibition of histone demethylase (KDM5), which would blunt an increase in activating histone marks (H3K4me3) whereas, succinate stabilizes the transcription factor, HIF-1a and induce IL-1β production.^38,39^ Additionally, the TCA cis-aconitase generates itaconate which has been shown to play an opposing role to the above by blocking TI-inducing tolerance.^40^ Together, the reprogramming of these metabolic pathways impacts epigenetic regulation of genes involved in immune responses driving TI.

### Molecular mechanism initiating EG

EG can be initiated by several distinct pathways. These pathways are reviewed below, with a focus on those where BCG is known to be relevant. Under homeostasis, hematopoietic stem cells (HSC) in the bone marrow (BM) constantly generate a limited number of blood cells, of which neutrophils account for up to 70% of white blood cells (leukocytes).^41^ EG is a hematopoietic response to severe infection, defined by heightened *de novo* generation of neutrophils. Such a response can be initiated by direct activation of HSC after detecting pathogens via PRR.^42^ Other cells such as endothelial cells can also stimulate EG indirectly by sensing the pathogen and secreting myelopoietic growth factors, primarily granulocyte-colony stimulating factor (G-CSF).^43^ The most pertinent example comes from the recent observation that a BCG-driven increase in G-CSF production was both necessary and sufficient to induce EG and that it was the increase in neutrophils that provided protection of neonates from sepsis.^11^ It remains unknown how and where BCG induces the production of G-CSF, but possible candidates include endothelial cells and keratinocytes recognizing components of BCG via PRRs.^44,45^ G-CSF engages the G-CSF receptor which is expressed on several myeloid lineages including early hematopoietic and progenitor cells (HPSCs), where it activates Janus kinase (JAK) and signal transducer and transcription activator (STAT-3). Upon activation, STAT3 is transported into the nucleus and upregulates the expression of the master transcriptional factor for EG, CCAAT/enhancer-binding protein beta (C/EBPβ), but not C/EBPα (the canonical marker of steady-state granulopoiesis (SE)).^46,47^ This was corroborated by the finding that BCG-mediated rapid EG induction depends on the upregulation of C/EBPβ, but not C/EBPα.^11^ Both the EG and SE transcription factor binding sites are enriched in the promoter of the *Myc* gene, which regulates the cell cycle and proliferation. STAT3 alone or coupled with C/EBPβ dissociates C/EBPα from the promoter region, inducing *Myc* expression and with that cell proliferation. This shift drives myeloid progenitors to EG, followed by differentiation into mature neutrophil populations.^46^

### Relationship of the molecular mechanism driving TI and EG

Following appropriate stimulation, cells exhibiting a trained immunity phenotype can be detected for months, i.e., beyond the short lifespan of the myeloid cells such as neutrophils and monocytes with approximately 19 hours or 7 days, respectively.^48,49^ This time course suggests that immune training may have occurred in long-lived precursors, e.g., at the level of HSC. BCG can persist in the body for perhaps years, continuing this process.^50–53^ However, interaction of BCG with the other commensal microbes could also contribute to this long-term training effect.^54,55^ Similar to the mechanism of EG, TI driven by BCG or β-glucan results in changes of cells in the BM that release mediators which in turn induce expansion of HSC and multipotent progenitor cells (MPP).^18,56^ Signaling through IFN-γ-IFN-γR induces not only expansion of the HSC, but also shifts production towards myeloid cells at the expense of the lymphoid lineage.^18,57^ Specifically, in mouse models BCG vaccination upregulates the expression of myeloid-lineage markers *(*Cebpe, Cebpa, IRF8, Csf1r, and Csf2rb) while the lymphoid-lineage markers (Lck, Rag1, Rag2, Pax5, and Irf4) are diminished in HSC and MPP, suggesting a shift towards myelopoiesis concomitant with the reduction of lymphopoiesis. Furthermore, the transcription factor (TF) hepatic nuclear factor 1A and B (HNF1A and HNF-1A) are essential for BCG-driven myeloid differentiation and TI in humans, whereas in the mouse model of LPSinduced TI, the TF was C/EBP, which, as mentioned above is also the main transcriptional regulator in BCG-induced EG.^11,58,59^ BCG-mediated induction of myelopoiesis in human infants confirmed enhanced expression of genes linked to myeloid, and in particular granulocytic cell lineage differentiation markers (CX3CR1, MPEG1, IRF4, C/EBPδ, A12 (S100A12), A9 (S100A9) within HSCs and MPPs after three months of vaccination.^58^

**Figure 1.**
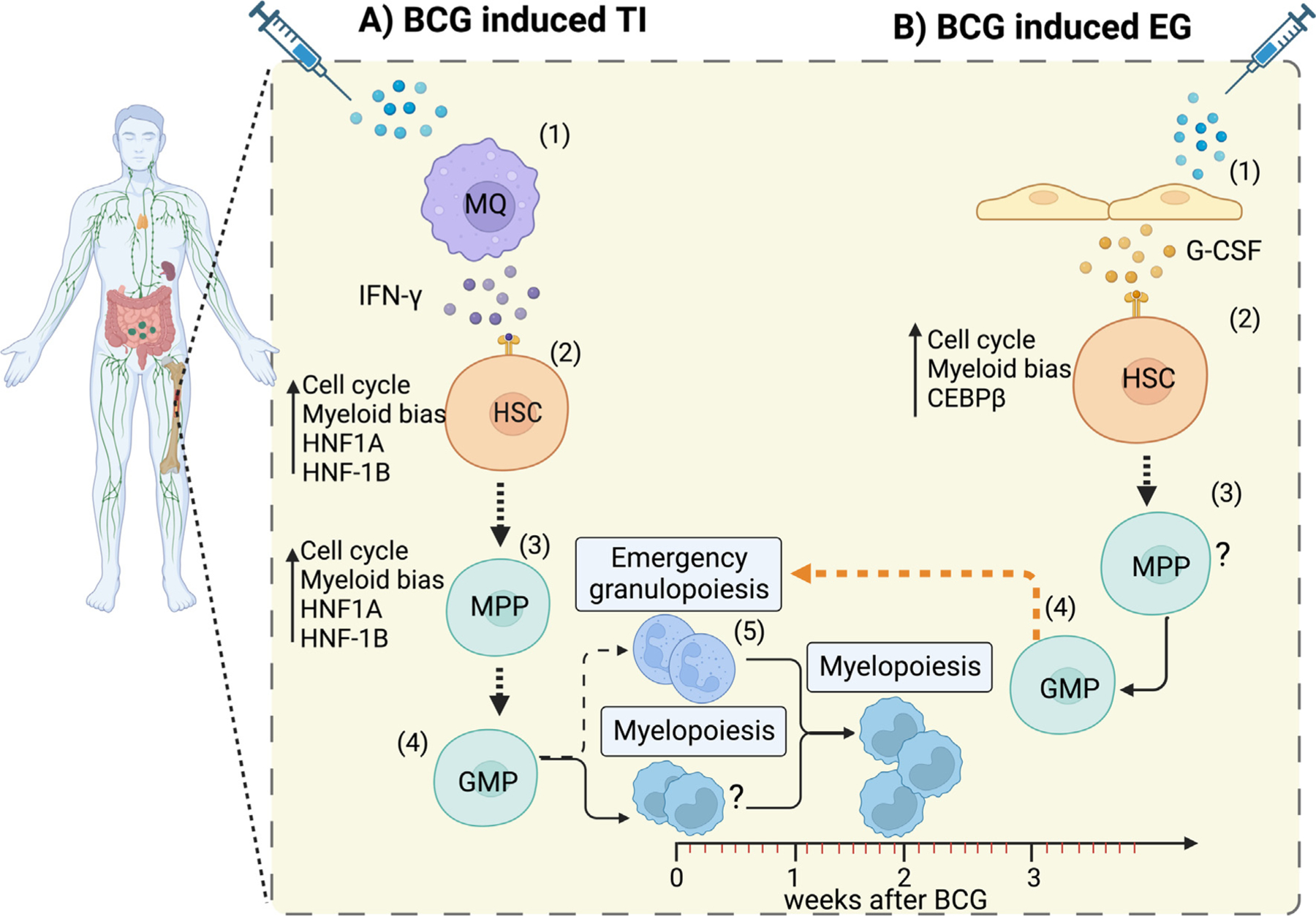
The relationship of the molecular mechanism driving TI and EG. **(A)** BCG vaccination activates macrophages or other cells (1) which release IFN-γ to expand and bias HSC to myelopoiesis (2). The expression of TF (HNF1A and HNF-1B) in HSC and MPP (3) induces TI characterized by the differentiation into GMP (4) and increased granulopoiesis in the early phases (5), followed by myelopoiesis. **(B)** BCG can also trigger the expansion of HSC and differentiation to myeloid progenitors in response to G-CSF produced by unknown cell type, likely endothelial cell (1) and upregulated expression of the canonical TF marker of EG (CEBPβ) in the bone marrow and spleen of newborns (2). As a result, in EG similar to TI, progenitors (3, 4) are biased towards enhanced granulopoiesis (EG) within three days (5). IFN; Interferon-gamma. MQ; Macrophage. HNF; Human Nuclear Factor. HSC; Hematopoietic stem cell. MPP; Multipotent progenitor. GMP; Granulocyte Monocyte progenitor. G-CSF; Granulocyte colony-stimulating factor. Created with BioRender.com. (Agreement number: LU252D4HAX)

Based on the above and coupled with the finding that the number of neutrophils is found to be higher in BCG-vaccinated vs -unvaccinated human newborns, suggests that the polarization of hematopoiesis following BCG administration towards granulopoiesis, i.e., EG, is likely one of the early steps for TI not just in newborn mice but also humans.^11,58^ It thus is entirely plausible to hypothesize that neutrophils are amongst the first responders in the early stages of TI across species. On this count, BCG induces TI expanded neutrophil numbers for at least two weeks in human recipients, that subsequently return to baseline levels by three months post-vaccination while maintaining long-term changes in functional reprogramming. In mice, this dynamic played out over 7 days, i.e. the same happened in mice as it did in humans, only faster.^15^ However, it should be noted that TI is not limited to granulopoiesis but affects myelopoiesis more broadly, e.g. includes generation of monocytes from the same progenitor cells (GMP).^18^ While the published literature has focused on mostly metabolic and epigenetic changes in monocytes undergoing TI, initiation of TI includes alteration of the HSC towards enhanced myelopoiesis which at least initially includes granulopoiesis.^41,58^ We thus postulate that the initial stages of BCG-induced TI involve enhanced neutrophil production via EG, i.e., that EG and TI in response to BCG are part of the same molecular process but simply capture different time points along a continuum of molecular events (Figure 1).

### Modulator of TI and EG

Given the importance of TI and EG for protection from infectious diseases, examining the range of possible modulators for these mechanisms appears prudent.

### Potential impact of age and metabolism on EG and TI

Newborns are more susceptible to suffering from infectious diseases.^60^ As mentioned earlier, vaccination with BCG at birth can reduce this risk via triggering pathogen-agnostic effects including EG.^2^ Clear evidence for TI in newborns is currently lacking. On the other hand, protection in association with TI has been observed in adults, where vaccination with BCG provides protection against experimental viral infection.^7,8,11^ Clear evidence for EG in adults is currently lacking. Possible age-dependent differences in TI or EG are thus currently not at hand. However, newborns and adults display differences in metabolism that might lead to age-dependent differences in the phenotypic manifestation of TI/EG. Immune cells including innate cells undergo metabolic alterations following the intensified bioenergetic demand upon detection of infectious invaders in order to eliminate them.^61,62^ In doing so, these cells dynamically switch on and off several metabolic pathways and employ various metabolic nutrients including glucose and FA to maintain effector responses (such as cytokine secretion) while ensuring the delivery of sufficient energy to several vital body organs.^63,64^ In newborns, such heightened energic cost interferes with growth and development.^65^ Unlike adults who can increase glucose and FA metabolism, the already high ratio of energy demand to energy reserves in neonates may contribute to the newborn’s tendency towards lower ‘energy cost’ of immune responses such as disease tolerance and with that increased susceptibility to suffer from infections.^64,66,67^ Specifically, comparing rates of glycolysis in LPS-stimulated monocytes from preterm vs term neonates and adults reveals a reduced glycolytic capacity that impacts effector cytokine production in newborns, though glycolysis in term monocytes was variably affected.^68^ This is further corroborated by reduced total protein expression as well as activation (phosphorylation) of mTOR (the activation of which upregulates glycolysis) and its upstream activator, RAC-alpha serine/threonine protein kinase in newborn as compared to adult macrophages. Circulating factors like alarmins (particularly increased expression of S100A8 and S100A9) in cord blood (CB) could contribute to the decreased mTOR expression in the newborns.^69^ However, the expression of several upstream and downstream regulators of mTOR including HIF-1α are found to be increased in LPS-triggered preterm monocytes as compared to adult monocytes, indicating that the regulatory cascades of metabolism in relation to TI and EG in newborn vs adult immune cells are currently insufficiently delineated.^68^ In addition to reduced glycolysis, transcriptional analysis of unstimulated preterm, term, and adult monocytes indicated distinct metabolic features by age and with a considerably diminished expression of genes corresponding to oxidative phosphorylation and FA metabolism in preterm monocytes.^68^ Thus, while age-dependent differences in metabolism could indicate a potential age-dependent difference in TI and EG, there currently is no data to corroborate or refute this concept.

### Impact of route on TI and EG

In animal models, the standard route of BCG vaccination (intradermal; ID) has long been challenged by superior protection through other alternative routes. In adult non-human primate (NHP) and mouse models, studies showed that ID immunization provides a relatively lower or no protection against *Mtb* infection compared with aerosol (AE), mucosal immunization (muc, i.e., endobronchially), as well as intramuscular (IM) or intravenous (IV) routes^19,70–73^ (Table 1). Furthermore, despite excellent protection against *Mtb* infection in the above-mentioned animal studies that in part appear attributable to the induction of robust CD4^+^ T cell responses, and in particular the presence of tissue-resident PD1^+^KLR^−^CD4^+^ T cells,^73^ these studies failed to detect a significant TI effect unless BCG was given IV. A recent experiment in mice supports this notion, as IV but not SC BCG vaccination induced off-target protection against SARS-CoV-2 through altering of innate immunity in the lung.^21^

**Figure 2.**
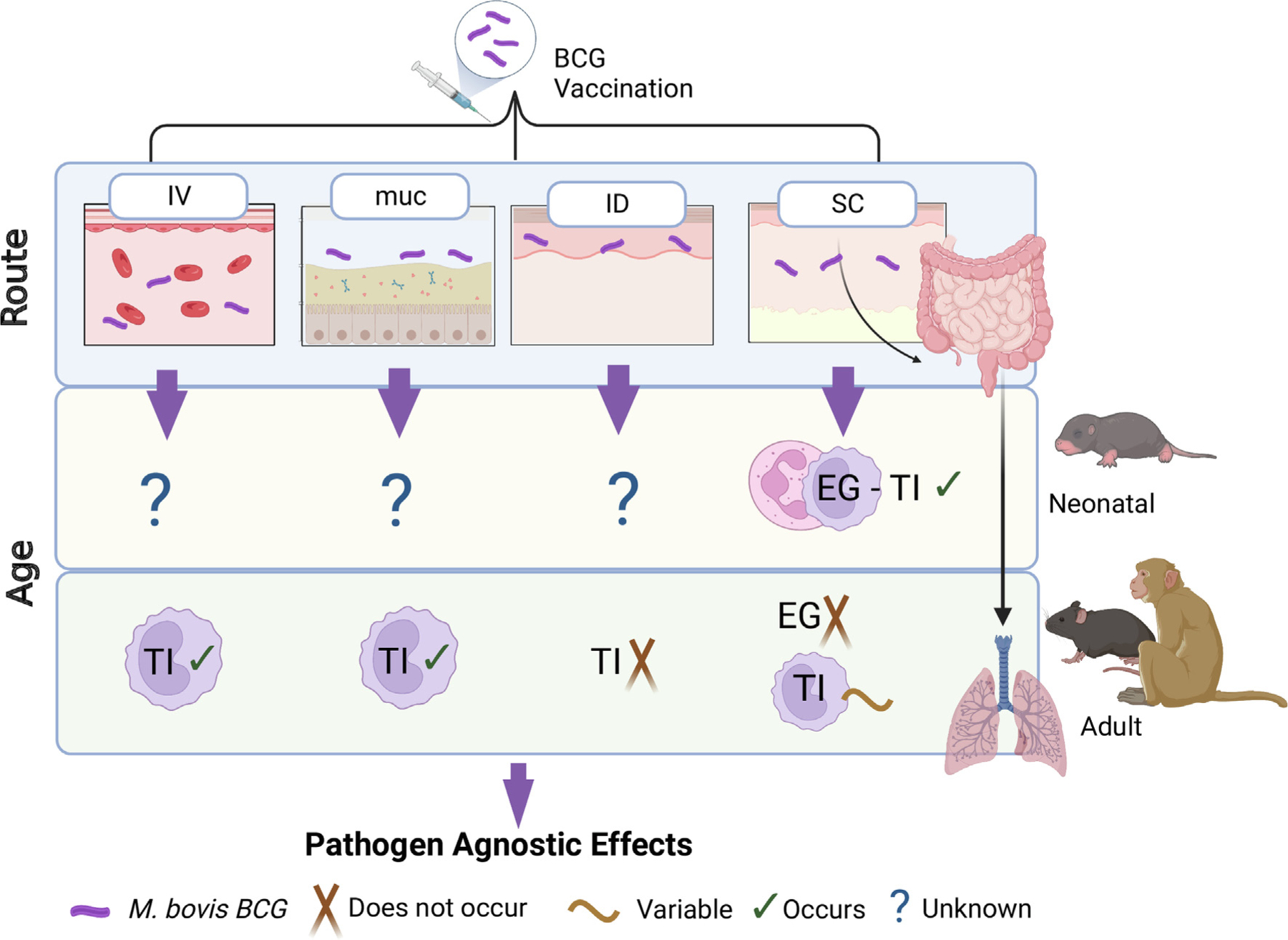
The impact of age and route of bcg vaccination on eg/ti. Following BCG immunization through either IV, muc, ID, or SC, *M. bovis* BCG initiates signaling cascades that induce either EG or TI. Induction of EG or TI is age dependent. Furthermore, SC delivery can also trigger TI in the lung, through the gut-lung axis mechanism where the change in the intestinal metabolites and microflora signals TI formation in the lung. Eventually, these circulating or resident cells mediate beneficial pathogen-agnostic effects. IV; Intravenous. ID; Intradermal. muc; mucosal. SC; Subcutaneous. EG; Emergency granulopoiesis. TI; Trained immunity. Created with BioRender.com. (Agreement number: EB25DTB78B).

Experimental evidence also supports profound induction of TI in adult rhesus macaque vaccinated via muc as compared with ID routes characterized by greater production of cytokines associated with TI (IL-1β, IL-6, and TNF-α) and increased aerobic glycolysis.^20^ Another study in adult mice showed that following BCG IV vaccination the number of HSC and MPP cells expanded in the BM, whereas BCG given SC did not induce such expansion. This study also noted that IV vaccination promoted myeloid bias in MPP cells (i.e., gene expression profile closely related to myeloid (MPP3), not lymphoid (MPP4) lineage), a bias that was not observed following BCG given SC (similar bias towards MPP3 and MPP4).^18^ Reprogrammed monocytes following IV BCG were more efficient in clearing Mtb in vitro, while no differences in clearance were observed when BCG was given SC. Reprogramming of myeloid progenitor cells and bias to GMP cells was however demonstrated following intraperitoneal (IP) injection of β-glucan in adult mice, as well as following BCG SC injection in a neonatal model.^11,56^ Notably, rewiring in HSC and subsequent induction of TI has been shown in ID BCG-vaccinated human adults^58^ (Table 2). Since the number of BCG bacteria found in the bone marrow positively correlates with expansion of HSC, one plausible explanation for greater induction of TI following IV administration over ID or SC is that BCG gains access to the BM in great numbers compared to the other routes.^18^ Overall, although in adult animal models different routes impact downstream induction of TI, the molecular mechanisms that determine this impact of the route have not been delineated. Importantly, none of the work on route of BCG administration has been conducted in newborn animals or humans, aside from evidence that BCG given SC expand myeloid progenitor in the BM and spleens of newborn mice, however, different routes were not directly compared in this study.^11^ Given the evidence that BCG when it was originally given orally and is now given ID has been shown to induce pathogen-agnostic protection in newborns suggests that the impact of route does not seem to be as a restrictive in newborns as it is in adults.^71,74,75^ Of interest in this context, intestinal alterations following SC BCG delivery generated TI in lung-resident macrophages, indicating the possible existence of a gut-lung axis of innate immune training. The intestinal dysbiosis modulated by parenteral BCG vaccination is associated with reduced cecum size, altered intestinal barrier, and an altered gut microbiome characterized by reduced richness and a greater relative abundance of operational taxonomic unit (OTU) assigned to the *Lactobacillaceae* family. These changes are further associated with an altered gut and lung metabolome and evidence for TI among immune cells in the lung.^76^ This hints at the existence of complex in vivo pathways where route of administration modulates the host in response to BCG (Figure 2).

## Conclusions

Pathogen-agnostic effects of vaccines contribute much to the impact of vaccines.^77^ Mechanisms providing pathogen-agnostic benefits following BCG vaccination at least in part relate to TI and EG. Current evidence suggests EG may be the initial phase of TI, i.e., they are part of the same process across time. Precise evidence regarding the molecular mechanisms by which BCG begins to induce EG/TI is lacking. With this, our understanding how BCG induces pathogen-agnostic protection is limited. It also remains unexplored how EG/TI differ across different hosts (age such as newborn vs adult, as well as metabolic state) and why BCG impact can vary depending on route of administration. Further, new TB vaccine candidates such as the MTBVAC vaccine (containing a live, attenuated strain of *M. tuberculosis*) can also induce trained immunity and offer non-specific protection against unrelated pathogens.^78^ Knowledge gained for BCG vaccination may therefore apply even if BCG is replaced with a vaccine that offers superior protection against TB disease. The current lack of knowledge, however, prevents optimal deployment of one of the most powerful, broad-spectrum anti-infectives ever made, the centenarian BCG.^79^ An unequivocal understanding of this will help to design future vaccines including TB and other pathogens.

## Figures and Tables

**Table 1:** The impact of route on TI/EG in animal models.

Type of animal	Sample size (N)	Age of animal	BCG strain	BCG dose	Route with greater protection	Trained immunity	Correlates of protection	Ref.
Indian-origin rhesus macaques	52 (10 ID_low_,8 ID_high,_ 10 IV, 10 AE, 10 AE/ID, and 4 Unvax	3–5 years	BCG Denmark	5 × 10^7^ CFUs (ID_high_, IV & AE), vs 5 × 10^5^ (ID_low_, and AE/ID)	IV	Not induced	Expanded lung resident T cells	Darrah et al. ^19^
Indian-origin rhesus macaques	27 (3 IV vs 12 muc vs 12 ID)	>4 years	Sofia (InterVax Ltd.)	5 × 10^5^ CFUs (IV, muc & ID)	Not determined	muc and IV	Not determined	Vierboom et al. ^20^
Indian/chines model of rhesus macaques	12 (6 ID vs 6 muc)	6–8 years	BCG Denmark	5 × 10^5^ CFUs (ID and muc)	muc	Not determined	Alteration of red cell induces (MCV, MCH) and monocyte/lymphocyte ratio	Verreck et al. ^71^
Indian-origin rhesus macaques	24 (8 ID vs 8 muc vs 8 Unvax)	4––7 years	Sofia (InterVax Ltd.)	1.5–6.0 × 10^5^ (ID and muc)	muc	Not determined	Local poly-functional Th17, IL-10 and IgA	Dijkman et al. ^80^
C57BL/6J Mice	8–12/group (SC vs IV)	6–10 weeks	BCG Pasteur	1 × 10^6^ CFUs (SC and IV)	Not determined	IV	IFN-γ signaling in macrophage	Kaufmann et al. ^18^
K18-hACE2	4–5/group (SC vs IV)	7–9 weeks	BCG Pasteur	1 × 10^6^ CFUs (SC and IV)	IV	Not determined	Restraining of innate immune responses	Hilligan et al. ^21^
BALB/C Mice	15 (7 ID vs 8 muc)	8 weeks	BCG Denmark	2 × 10^5^ CFUs (ID & muc)	muc	Not determined	Lung resident CD4 T cells exhibiting PD-1^+^KLRG^−^	Bull et al. ^73^

Abbreviations:-ID: Intradermal; muc: Intramucosal; IV: Intravenous; SC: Subcutaneous; AE: Aerosol; Unvax: Unvaccinated control; MCV: Mean Corpuscular volume; MCH: Mean Corpuscular hemoglobin; Ref.: references.

**Table 2:** BCG vaccination in Humans.

Study area	Sample size (N)	Age	BCG strain	Route	Mechanism of Trained immunity	Immune response	Ref.
Netherlands	15	19–37 years	BCG Denmark	ID	Reprogramming of monocytes	IL-1β with viral infection	Arts et al. ^8^
Netherlands	15	19–50 years	BCG Bulgaria	ID	Expansion of HSC and Long-term macrophage	Not determined	Cirovic et al. ^58^
Netherlands	25	20–70 years	BCG Bulgaria	ID	Expansion of HSC and Long-term neutrophil	Not determined	Moorlag et al. ^15^
Netherlands	10	18–35 years	BCG Bulgaria	ID	Activation of NK cell and monocytes	Activation of NK cells and monocytes with malaria	Walk et al. ^9^
Guinea Bissau	60	At birth	BCG Denmark	ID	Changes in concentrations of plasma lysophospholipids that correlated with cytokine response	Not determined	Diray-Arce et al. ^81^
Netherlands	303	17–71 years	BCG Bulgaria	ID	Plasma metabolites determines BCG induced TI	Not determined	Koeken et al. ^82^
Papua Guinea vs Gambia	6–8/group	At birth	BCG Denmark	ID	Not determined	EG	Brook et al. ^11^
United Kingdom	8	At birth	BCG Denmark	ID	Not determined	Expansion of Th17 (CD4^+^IL17^+^) cells	Schaltz-Buchholzer and Toldi ^83^

**Abbreviations:** ID: Intradermal; IL-1β: Interleukin 1β; EG: Emergency Granulopoiesis.
